# Combination therapy with F5/35 fiber chimeric conditionally replicative adenoviruses expressing IL‐24 enhances the antitumor effect of temozolomide against melanoma

**DOI:** 10.1002/cam4.1843

**Published:** 2018-11-08

**Authors:** Ming Yang, Chunsheng Yang, Yingkai Tao, Jianqin Tang, Qian Huang, Wenwen Guo, Shouxin Feng, Aijun Jiang, Xifeng Xu, Guan Jiang, Yanqun Liu

**Affiliations:** ^1^ Department of Dermatology Affiliated Hospital of Xuzhou Medical University Xuzhou China; ^2^ Department of Radiotherapy Affiliated Hospital of Xuzhou Medical University Xuzhou China; ^3^ Department of Dermatology, The Second People’s Hospital of Huai’an Affiliated Huai’an Hospital of Xuzhou Medical University Huai’an China

**Keywords:** conditionally replicative adenovirus, CRAd, F5/35, interleukin 24, melanoma, temozolomide

## Abstract

**Background:**

Temozolomide (TMZ) is widely used to treat melanoma; however, response rates to TMZ are low because of rapid and frequent resistance. Conditionally, replicative adenoviruses (CRAds) are an effective and promising approach. The receptor for adenovirus is coxsackie‐adenovirus receptor (CAR), which is poorly expressed in most cells. However, CD46, which is the receptor of species B adenoviruses (Ads), is highly expressed in many cells.

**Methods:**

We constructed CRAd F5/35‐ZD55‐IL‐24, which uses the viral receptors CAR and CD46 for entry into cells. We investigated the antitumor effect of F5/35‐ZD55‐IL‐24 in combination with TMZ to treat melanoma in vitro and in vivo.

**Results:**

The \results indicated that F5/35‐ZD55‐IL‐24 in combination with TMZ produced additive or synergistic antitumor and pro‐apoptotic effects in melanoma cells. The combination of F5/35‐ZD55‐IL‐24 and TMZ significantly inhibited the growth of melanoma in vivo. In addition, the antitumor effect of F5/35‐ZD55‐IL‐24 was superior to that of ZD55‐IL‐24 and ZD55‐IL‐24 combined with TMZ.

**Conclusions:**

The use of F5/35‐ZD55‐IL‐24 in conjunction with TMZ is a promising approach for anti‐melanoma therapy.

Our results indicated that F5/35‐ZD55‐IL‐24 in combination with TMZ produced additive or synergistic antitumor effect and pro‐apoptotic effect in melanoma cells highly expressed CD46. The combination of F5/35‐ZD55‐IL‐24 and TMZ significantly inhibited the growth of melanoma in vivo. We also found the antitumor effect of F5/35‐ZD55‐IL‐24 was superior to ZD55‐IL‐24, the combination of F5/35‐ZD55‐IL‐24 and TMZ had a more significant antitumor effect than ZD55‐IL‐24 combining with TMZ.

## 1 INTRODUCTION

Melanoma, a skin cancer with an extremely high mortality rate, is a serious health concern both in the United States and worldwide,^1^ and its incidence is increasing.^2^ Recently, new therapies such as molecular‐targeted therapy (BRAF inhibitors^3^ and MEK inhibitors^4^), immunotherapy (Ipilimumab^5^ and PD‐1 inhibitors^6^) or oncolytic virus therapy (talimogene laherparepvec,^7^ the only oncolytic virus having gained FDA approval for melanoma treatment, which is designed to induce lysis of tumor cells) have emerged. These treatments exhibit unique advantages, fewer side effects, and a higher patient survival rate. Unfortunately, the heterogeneity and multiple drug resistance of tumors have restricted the development of therapeutics.^8^ Recently, Jason et al reported that talimogene laherparepvec in combination with ipilimumab demonstrated significantly higher response rates.^9^ In addition, a combination of anti‐CD25 and PD‐1 inhibitors appeared to show a synergistic antitumor effect.^10^ Combination therapy may represent a promising strategy to overcome the obstacles encountered in oncotherapy.

Dacarbazine (DTIC), an alkylating agent, has been the approved first‐line treatment for metastatic melanoma in routine clinical practice. Temozolomide (TMZ) is an oral chemotherapy with good bioavailability,^11^ whose metabolically active form is 5‐(3‐methyl)‐1‐(triazen‐1‐yl) imidazole‐4‐carboxamide (MTIC) and the activated form is DTIC.^12^ TMZ has only limited success in the treatment of melanoma because of resistance^13^; therefore, a new approach is required to sensitize melanoma cells to TMZ.

One effective and novel approach in the treatment of malignant tumors is the use of conditionally replicating adenoviruses (CRAds).^14^ CRAds not only augment antitumor activity by transducing therapeutic genes into cancer cells, but also selectively lyse tumor cells, without affecting normal cells.^15^ Their antitumor effect is augmented when combined with chemotherapy or radiation therapies.^16^ CRAds are obtained by deleting the genes that are essential for their activity and replication in normal cells but are not necessary for tumor cells. Examples include ZD55 with a deleted E1B‐55 K gene^17^ and delivering the gene required for viral replication using a tumor‐specific promoter.^18^

ZD55 vectors are commonly derived from adenovirus serotype 5 (Ad5), which binds to the coxsackie‐adenovirus receptor (CAR) on the cell membrane.^19^ Twelve fiber trimers of Ad5 are attached to the penton bases on the capsid. Each fiber monomer ends in a globular region known as the knob domain, which is associated with cellular adenoviral receptors. Replication of Ad5 in many primary tumor cells in vivo is often limited because of low or absent expression of CAR.^20^ Thus, non‐CAR receptors from other Ad serotypes, such as Ad serotype 35 (Ad35), are used instead. Ad35 enters cells via CD46, a non‐CAR receptor widely expressed in most tumor cells.^21^

Interleukin‐24 (IL‐24) is a member of the IL‐10 cytokine family. Recent evidence suggests IL‐24 is a promising candidate for cancer gene therapy. The expression of IL‐24 from replication‐defective adenovirus carrying IL‐24 (Ad‐IL‐24) can suppress cancer cell growth and induce apoptosis in a variety of cancer cells.^22^ Li et al^23^ found that IL‐24 has a novel anticancer effect toward oral squamous cell carcinoma cells and that autophagy inhibition could improve the anticancer effect of IL‐24.

In the present study, we synthesized a fiber chimeric adenoviral, termed F5/35‐ZD55‐IL‐24. The fiber protein of serotype 5 adenovirus (Ad5) was substituted by that of serotype 35 adenovirus (Ad35). The antitumor activity of F5/35‐ZD55‐IL‐24 combined with TMZ in A375 and MV3 cells was investigated. Moreover, the synergistic effect of combined treatment was evaluated in comparison with virotherapy or TMZ treatment alone. In addition, fluorescence‐activated cell sorting (FACS) analysis, immunohistochemistry, and Western blotting were used to test the apoptotic effect of the F5/35‐ZD55‐IL‐24 with TMZ. The results indicated that this vector is a promising strategy for selectively transferring genes into human melanoma cells.

## 2 MATERIALS AND METHODS

### 2.1 Cell lines, TMZ, and virus synthesis

#### 2.1.1 Cell culture and reagents

Two human melanoma cell lines (A375 and MV3) were purchased from the Chinese Academy of Sciences (Shanghai, China) and Nanjing Keygen Biotech (Nanjing, China), along with normal human lung fibroblasts (the Chinese Academy of Sciences). The cell culture medium was Dulbecco's Modified Eagle Medium (DMEM; Gibco, Carlsbad, CA, USA) supplemented with 10% heat‐inactivated fetal bovine serum (Sijiqing, Zhejiang, China), 4 mmol/L glutamine, 50 U/mL penicillin, and 50 μg/mL streptomycin. Cells were cultured at 37°C in a humidified atmosphere with 5% CO_2_. Regular screening during the log phase of growth confirmed the lack of mycoplasma contamination.

#### 2.1.2 Preparation of TMZ

A 5 mL volume of 3,4‐Dihydro‐3‐methyl‐4‐oxoimidazo[5,1‐d]‐as‐tetrazine‐8‐carboxamide (TMZ; Schering‐Plough, Osaka, Japan) was dissolved in dimethyl sulfoxide (DMSO; Sigma, St. Louis, MO, USA) and used at concentrations ranging from 0 to 800 μmol/L.

#### 2.1.3 Adenovirus construction

The shuttle plasmids pZD55 or pZD55‐IL‐24 were co‐transfected into HEK293 cells using either the adenoviral backbone plasmid pBHGE3 (Microbix Biosystems Inc, Mississauga, ON, Canada) or a fiber chimeric adenoviral backbone plasmid pF5/35 (a gift from Professor Changqing Su, The Second Military Medical University, China). Lipofectamine 2000 (Invitrogen, Waltham, MA, USA) was used as the transfection agent, according to the manufacturer's instructions. Plaques were observed at 9‐14 days post‐transfection. PCR was used to identify the transgenes using primers specific for *IL24*. The adenoviruses were grown in HEK293 cells for amplification and titration via the 50% tissue culture infectious dose (TCID50). Virus stocks were maintained in an adenoviral buffer (10 mmol/L Tris‐HCl, pH 8.0, 2 mmol/L MgCl_2%_, and 4% sucrose).

#### 2.1.4 Infectivity of F5/35‐ZD55‐EGFP

A375 and MV3 cells were seeded in six‐well plates (at 3 ×  10^5^ cells/well) and treated with either ZD55‐EGFP (multiplicity of infection [MOI] 20) or F5/35‐ZD55‐EGFP (MOI 20). Expression of the enhanced green fluorescent protein (EGFP) was detected using an Olympus fluorescence microscope (Olympus, Tokyo, Japan) at 24, 48, and 72 hours after treatment.

### 2.2 Western blotting analysis

A375 and MV3 cells were treated with phosphate‐buffered saline (PBS), ZD55‐IL‐24 (MOI 20), F5/35‐ZD55‐IL‐ 24 (MOI 20), TMZ (100 µm/L), ZD55‐IL‐24 (MOI 20) + TMZ (100 µm/L), or F5/35‐ZD55‐IL‐24 (MOI 20) + TMZ (100 µm/L). Cells were removed from the plates and cell extracts were separated on a 12% SDS‐polyacrylamide gel. Proteins were transferred on to a nitrocellulose membrane and incubated at 4°C overnight, together with the following primary antibodies: rabbit anti‐CAR, rabbit anti‐CD46 (dilution 1:500; Santa Cruz Biotechnology, Santa Cruz, CA, USA); rabbit anti‐IL‐24 (dilution 1:500; Bioss, Beijing, China); mouse anti‐Bcl‐2, rabbit anti‐Bax, rabbit anti‐Pro‐caspase‐3, and mouse anti‐p53 (dilution 1:1000; Absci, College Park, MD, USA); rabbit anti‐MGMT (dilution 1:1000; Abcam, Cambridge, MA, USA); mouse anti‐E1A (dilution 1:500, EMD Millipore, Burlington, MA, USA); or rabbit anti‐β‐actin (dilution 1:5000; Bioworld, St Louis Park, MN, USA). The membranes were then washed and incubated for 2 hours with alkaline phosphatase‐conjugated secondary antibodies (goat anti‐rabbit, dilution 1:1000, Zhongshan, Beijing, China; goat anti‐mouse, dilution 1:1000, Zhongshan) in Tris‐buffered saline with Tween‐20 (TBST) and developed using a nitro‐blue tetrazolium and 5‐Bromo‐4‐chloro‐3' prime symbol‐indolyl phosphate (NBT/BCIP) color development substrate (Promega, Madison, WI, USA).

### 2.3 Quantitative real‐time PCR

Cells treated with adenovirus and untreated cells were both harvested to extract total RNA. The RNA was reverse transcribed into cDNA using the PrimeScript II reverse transcriptase kit (Takara, Dalian, China) according to the manufacturer's instructions. Real‐time PCR amplification of *IL24* was carried out under the following reaction conditions: 5 minutes at 95°C; followed by 40 cycles at 95°C for 10 seconds, 60°C for 34 seconds, 95°C for 15; annealing at 60°C for 1 minute finally, and 95°C for 15 seconds. PCR was performed using an ABI PRISM 7500 Sequence Detection System (Foster City, CA, USA) with *GAPDH* as the internal control. The sequences of the primers used were as follows: IL‐24, forward, 5‐GTACTCGAGATGAATTTTCAACAGAGG‐3 and reverse, 5‐ATGGATCCTGAGAGCTTGTAGAATTTCTGC‐3; GAPDH, forward, 5‐AATCCCATCACCATCTTCC‐3 and reverse, 5‐CATCACGCCACAGTTTCC‐3.

### 2.4 Immunocytochemical staining

Treatment consisted of ZD55‐IL‐24 (MOI 20), F5/35‐ZD55‐IL‐24 (MOI 20), TMZ (100 µm/L), ZD55‐IL‐24 (MOI 20) + TMZ (100 µm/L), or F5/35‐ZD55–IL‐24 (MOI 20) + TMZ (100 µm/L). After 48 hours, A375 and MV3 cells were fixed onto glass coverslips with 4% paraformaldehyde. After washing with PBS, cells were incubated with rabbit anti‐IL‐24 antibody (dilution 1:100; Bioss) for 24 hours. The cells were then incubated with horseradish peroxidase‐conjugated secondary antibody for 1 hour followed by colorimetric detection with diaminobenzidine. To evaluate the IL‐24‐positive fractions, the mean number was determined from at least 200 cells counted from six different regions.

### 2.5 Evaluation of the apoptotic effect of F5/35‐ZD55‐IL‐24 in conjunction with TMZ

Melanoma cells were treated using a previously published method.^24^ After 72 hours, the cells were stained with annexin V‐fluorescein isothiocyanate (FITC) and propidium iodide (PI), and then apoptosis was measured using an annexin V‐FITC apoptosis detection kit (Keygen Biotech). A375 and MV3 cells were seeded in six‐well plates (at 3 × 10^5^ cells/well) and treated with PBS, TMZ (100 µm/L), ZD55‐IL‐24, F5/35‐ZD55‐IL‐24, and virus in combination with TMZ (100 µm/L), all at an MOI of 20 for 48 hours. Cells were then harvested and washed twice with cold PBS. The prepared cells were resuspended in binding buffer (10 mmol/L HEPES/NaOH [pH 7.4], 140 mmol/L NaCl and 2.5 mmol/L CaCl_2_) at a concentration of 1 × 10^6^ cells/mL. Next, 5 mL annexin V‐FITC (Pharmingen, San Diego, CA, USA) and 5 mL of PI were added to the cells, which were then analyzed using a FACStar flow cytometer (Becton Dickinson, San Jose, CA, USA).

### 2.6 Apoptotic cell staining

Cells were then seeded in flat‐bottomed 24‐well plates with PBS, TMZ (100 µm/L), ZD55‐IL‐24, F5/35‐ZD55‐IL‐24, and virus in combination with TMZ (100 µm/L), all at an MOI of 20. Untreated cells served as controls. After treatment for 48 hours, cells were incubated in Hoechst 33258 (Keygen) for 10 minutes, washed twice in PBS, and observed under a fluorescence microscope (Olympus).

### 2.7 In vivo antitumor assay

Forty‐two male BALB/c nude mice (4‐5 weeks old) were purchased from Beijing dimension (Beijing, China). Animal welfare and the experimental procedures were carried out strictly in accordance with the ‘‘Guide for the Care and Use of Laboratory Animals.”^25^ When the mice were fed for 1 week under the condition of constant temperature and humidity, A375 (4 × 10^6^, 100 µL) cells were injected subcutaneously into the right back of the mice. When tumor volume reached 200 mm^3^, the mice were randomly divided into six groups, each group received 7.0 × 10^9^ PFU of virus and 100 mg/kg TMZ were injected intratumorally for three consecutive days. The tumor was measured every 5 days, and tumor volume was calculated by the following equation: *V* (mm^3^) = length × width2/2. After 25 days of treatment, the mice were sacrificed. Tumors were excised and weighed. The inhibition proportion of the tumor volume and the inhibition proportion of the tumor weight were calculated according to the following equation: [1 − (mean tumor volume in the control group − mean final tumor volume in the treatment group)/(mean tumor volume in the control group − mean final tumor volume in the control group)] × 100% and (mean tumor weight in the control group − mean tumor weight in the treatment group)/mean tumor weight in the control group × 100%.

### 2.8 In vivo immunohistochemistry

Immunohistochemistry was performed using a standard streptavidin‐peroxidase (Sp) kit (Zhongshan Biotech, Zhongshan, China). After 2 hours of dewaxing, tumor tissue slices were soaked in xylene for 20 minutes. Monoclonal rabbit anti‐IL‐24 antibodies (1:250), monoclonal rabbit anti‐MGMT antibodies (1:300), monoclonal rabbit anti‐Bcl‐2 antibodies (1:300), and monoclonal rabbit anti‐bax antibodies (1:300) were incubated with the slices overnight at 4°C; then a brown precipitate was generated using diaminobenzidine (DAB; Zhongshan Biotech). Hematoxylin was used to stain the nuclei.

### 2.9 Statistical analysis

Data were expressed as the mean ± standard deviation (SD) and differences between groups were analyzed using either an independent samples *t* test or ANOVA as appropriate, using SPSS Base 13.0 (SPSS Inc, Chicago, IL, USA). *P* values <0.05 were considered to indicate statistical significance.

## 3 RESULTS

### 3.1 Expression of CAR and CD46 varies in melanoma cancer cells

**Figure 1:**
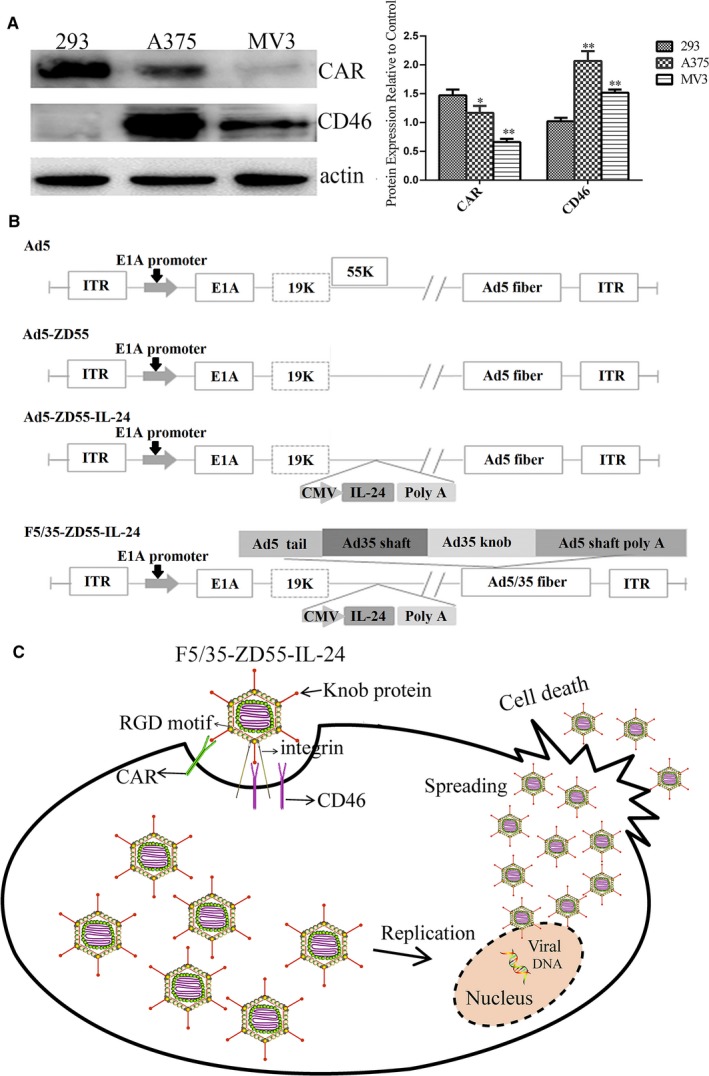
The expression levels of CAR and CD46 in melanoma cells and schematic diagrams of the adenovirus vectors and the mechanism of the novel chimeric adenovirus. A, Western blotting was performed to measure expression levels of CAR and CD46. β‐actin was used as a loading control. *P < 0.05 and **P < 0.01 vs 293 cells. B, Ad5, Ad5‐ZD55, Ad5‐ZD55‐IL‐24, and F5/35‐ZD55‐IL‐24. Ad5‐ZD55 includes an E1B‐55 K deletion. F5/35 fiber: 5 and 35 type adenoviral chimeric fiber. ITR: inverted terminal repeats. Ad5‐ZD55‐EGFP and F5/35‐ZD55‐EGFP (not shown) have the same structure as Ad5‐ZD55‐IL‐24 or F5/35‐ZD55‐IL‐24, with IL‐24 replaced by EGFP. C, F5/35‐ZD55‐IL‐24 firstly attaches to cells via CD46 or CAR. Once attached, the RGD motif in the penton base interacts with integrin, promoting endocytosis, and internalization of the adenovirus. The tumor cells are then lysed by F5/35‐ZD55‐IL‐24 via apoptosis. The released adenovirus infects adjacent tumor cells. CAR, coxsackie‐adenovirus receptor

CAR and CD46 are the primary cellular attachment receptors for Ad5 and Ad35, respectively. The expression of CAR in both melanoma cell lines was lower than that in HEK293 cells (*P* < 0.05), while the expression of CD46 was higher in both melanoma cell lines (*P* < 0.05) (Figure 1A).

### 3.2 High infectivity of F5/35‐ZD55‐EGFP in melanoma cells

**Figure 2:**
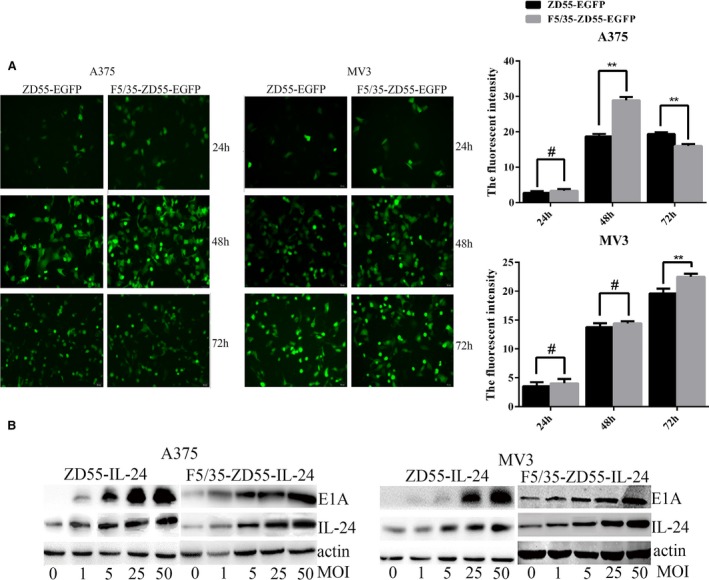
F5/35‐ZD55 shows high infectivity and efficient delivery of IL‐24 in A375 and MV3 cells. A, Expression of EGFP was detected using an Olympus fluorescence microscope at 24, 48, and 72 hours following treatment with ZD55‐EGFP (MOI 20) and F5/35‐ZD55‐EGFP (MOI 20) (scale bar, 50 μm). B, E1A and IL‐24 protein levels were assessed using Western blotting with the indicated antibodies to detect the infectivity of F5/35‐ZD55. β‐actin served as a loading control

The high expression of CD46 in melanoma cells indicated that Ad35 is a suitable gene vector. Therefore, we synthesized a 5/35 chimeric fiber adenoviral vector to transfect the therapeutic gene into the melanoma cell lines. Schematic diagrams of 5/35, the chimeric fiber adenovirus F5/35‐ZD55‐EGFP, and F5/35‐ZD55‐IL‐24, are shown in Figure 1B,C, indicating the mechanism of F5/35‐ZD55‐IL‐24. Figure 2A shows that the fluorescence intensity in A375 cells was the strongest at 48 hours, and the fluorescence intensity in the F5/35‐ZD55‐EGFP group was significantly higher than that in ZD55‐EGFP group (*P* < 0.05). However, at 72 hours, the fluorescence intensity in the F5/35‐ZD55‐EGFP group was lower than that in ZD55‐EGFP group (*P* < 0.05), which was because some A375 cells were dead. In MV3 cells, the fluorescence intensity in the F5/35‐ZD55‐EGFP group was significantly higher than that in the ZD55‐EGFP group at 72 hours (*P* < 0.05); however, there was no difference between the F5/35‐ZD55‐EGFP group and ZD55‐EGFP group at 24 and 48 hours (*P* > 0.05, Figure 2A). Taken together, these results indicated that the F5/35 chimeric fiber adenovirus had higher infectivity in A375 and MV3 cells than the Ad5 virus.

### 3.3 IL‐24 expression is mediated by F5/35‐ZD55‐IL‐24 in melanoma cells

We investigated the efficiency of F5/35‐ZD55‐mediated delivery of IL‐24 in both A375 and MV3 cells treated with ZD55‐IL‐24 or F5/35‐ZD55‐IL‐24. F5/35‐ZD55‐IL‐24 induced higher expression of IL‐24 than did ZD55‐IL‐24 in melanoma cells (Figure 2B) and showed high infectivity of melanoma cells in a vector dose‐dependent manner.

### 3.4 TMZ does not affect the replication of F5/35‐ZD55‐IL‐24 in melanoma cells

**Figure 3:**
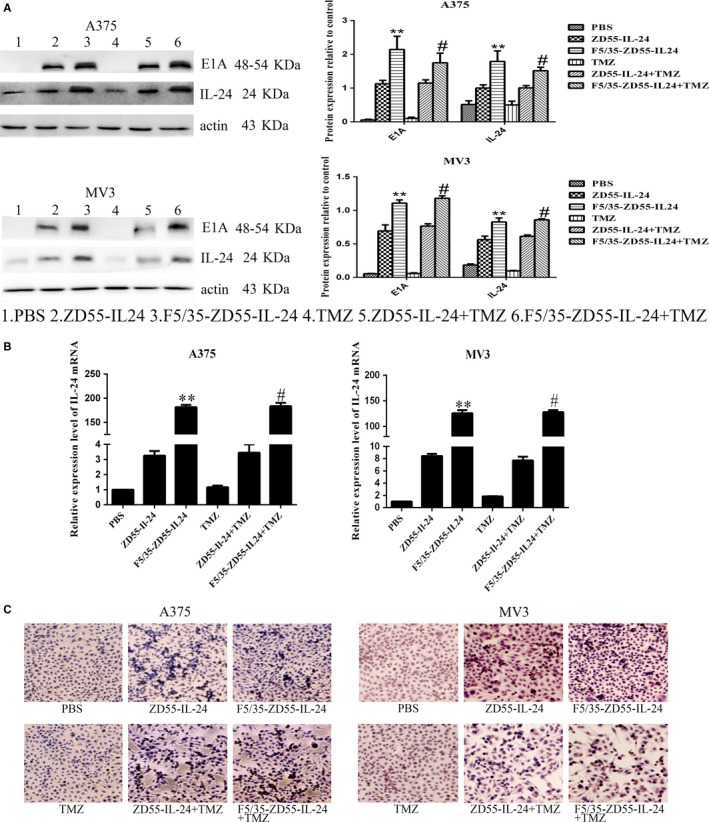
TMZ does not affect the replication of F5/35‐ZD55‐IL‐24 in melanoma cells. A, E1A and IL‐24 protein levels in infected cells were determined by Western blotting. β‐actin was used as the loading control. B, Expression levels of *IL24* mRNA were quantified using qRT‐PCR *GAPDH* was used as the loading control. C, IL‐24 protein was detected by immunocytochemical staining (scale bar, 50 μm. *P < 0.05 and **P < 0.01 vs ZD55‐IL‐24; # *P* > 0.05 vs F5/35‐ZD55‐IL‐24. TMZ, temozolomide

We found that F5/35‐ZD55‐IL‐24 could efficiently deliver IL‐24 to melanoma cells. To further explore the effect of TMZ on F5/35‐ZD55‐IL‐24‐mediated delivery of IL‐24 into melanoma cells, A375 and MV3 cells were treated with ZD55‐IL‐24, F5/35‐ZD55‐IL‐24, TMZ, ZD55‐IL‐24 + TMZ, or F5/35‐ZD55‐IL‐24 + TMZ. After treatment for 48 hour, IL‐24 showed stable overexpression in both A375 and MV3 cells. The level of IL‐24 in cells treated with F5/35‐ZD55‐IL‐24 was higher than that in cells treated with ZD55‐IL‐24 (*P* < 0.05). However, there was no significant difference in the IL‐24 level between the F5/35‐ZD55‐IL‐24 and F5/35‐ZD55‐IL‐24 + TMZ groups (*P* > 0.05) (Figure 3A). These results showed that F5/35‐ZD55 could induce a higher and more stable expression of IL‐24, and that TMZ did not affect IL‐24 expression. We found that there was a similar level of the adenoviral E1A protein between the F5/35‐ZD55‐IL‐24 group and the F5/35‐ZD55‐IL‐24 + TMZ group (Figure 3A), which indicated that F5/35‐ZD55‐IL‐24 replicated effectively in melanoma cells and that TMZ did not increase the infectivity of CRAds. We further detected the expression level of *IL24* mRNA by qRT‐PCR (Figure 3B) and the protein level by immunocytochemical staining (Figure 3C), both of which were consistent with the Western blotting results.

### 3.5 Enhanced apoptotic effect of F5/35‐ZD55‐IL‐24 in conjunction with TMZ

**Figure 4:**
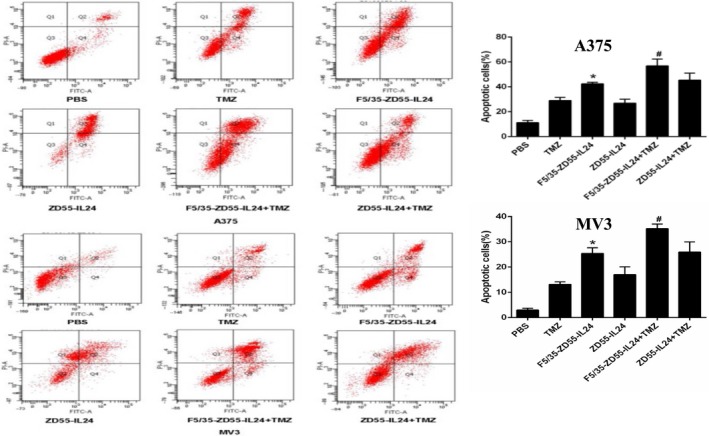
Induced apoptotic changes examined by Annexin V‐FITC staining. The mean percentage of cell apoptosis was calculated based on three independent experiments. *P < 0.05 vs ZD55‐IL‐24; #*P* < 0.05 vs F5/35‐ZD55‐IL‐24

To explore whether the apoptotic effect of F5/35‐ZD55‐IL‐24 was enhanced by TMZ, we used FACS analysis to examine apoptotic changes in melanoma cells. The combined treatment of F5/35‐ZD55‐IL‐24 with TMZ resulted in significantly higher levels of apoptosis than that seen in the other groups (*P* < 0.05, Figure 4).

**Figure 5:**
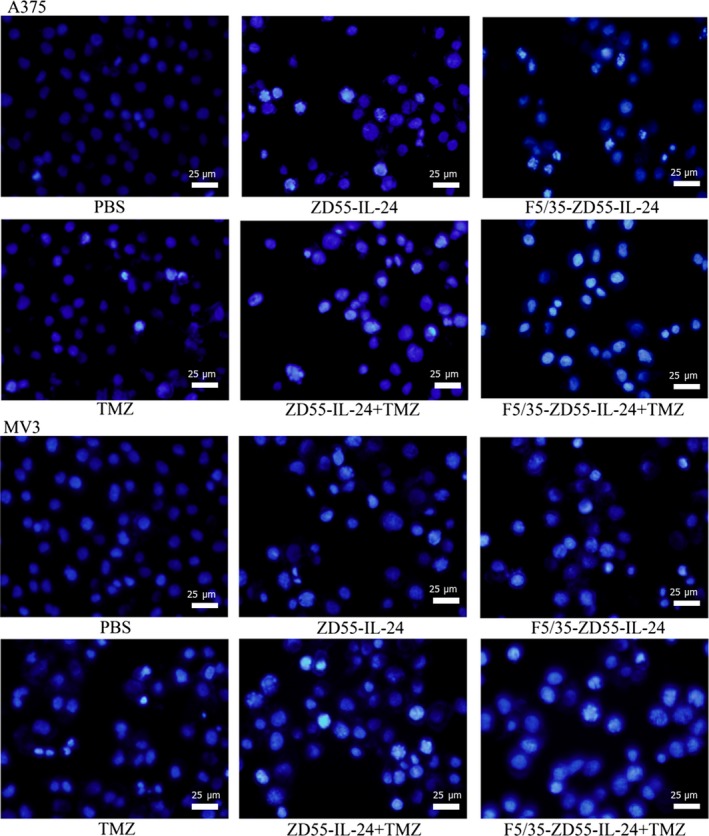
Morphological changes of the nuclei examined by Hoechst 33258 staining. Original magnification, 400× (scale bar, 25 μm)

Cell staining was used to detect the apoptotic effect of F5/35‐ZD55‐IL‐24 in conjunction with TMZ in melanoma cells. Consistent with the results from the FACS analysis, we found that nuclear morphology changes, including DNA fragmentation and chromatin condensation, was most prominent in the group treated with F5/35‐ZD55‐IL‐24 in conjunction with TMZ (Figure 5). These results suggested that TMZ acts synergistically with F5/35‐ZD55‐IL‐24 to increase cell apoptosis.

### 3.6 The effect of F5/35‐ZD55‐IL‐24 in conjunction with TMZ on apoptotic proteins in melanoma cells

**Figure 6:**
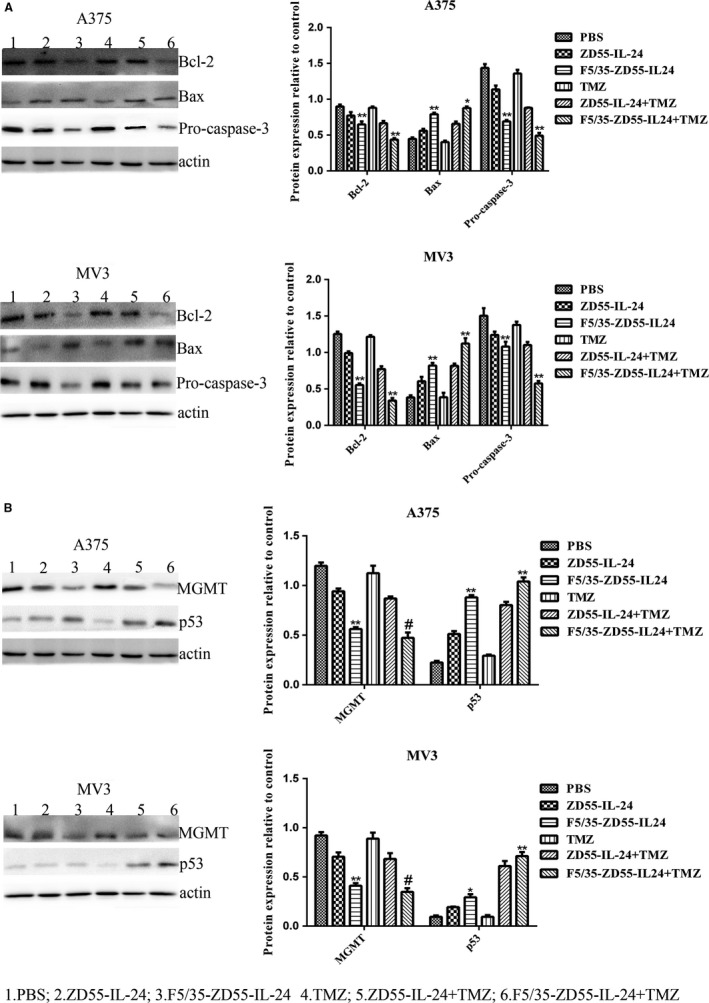
F5/35‐ZD55‐IL‐24 in combination with TMZ changed the levels of apoptotic proteins, MGMT, and p53. A, The levels of Bcl‐2, Bax, and Pro‐caspase‐3 were detected using Western blotting analysis. β‐actin was used as a loading control. B, p53 and MGMT protein levels were analyzed using Western blotting with anti‐p53 and anti‐MGMT antibodies. β‐actin was used as a loading control. *P < 0.05 and **P < 0.01, F5/35‐ZD55‐IL‐24 vs ZD55‐IL‐24, F5/35‐ZD55‐IL‐24 + TMZ vs F5/35‐ZD55‐IL‐24; #*P > *0.05 vs F5/35‐ZD55‐IL‐24. TMZ, temozolomide

We investigated whether expression of apoptotic proteins was influenced by F5/35‐ZD55‐IL‐24 in conjunction with TMZ. A375 and MV3 cells were treated with PBS, ZD55‐IL‐24, F5/35‐ZD55‐IL‐24, TMZ, ZD55‐IL‐24 + TMZ, or F5/35‐ZD55‐IL‐24 + TMZ. After 48 hours, we detected the protein levels of Bcl‐2, Bax, and caspase‐3 using Western blotting. We observed decreased levels of Bcl‐2 and caspase‐3 in both groups treated with the virus alone; however, the change was more significant in cells treated with F5/35‐ZD55‐IL‐24 (*P* < 0.05). The levels of the apoptotic proteins were further reduced by the addition of TMZ, especially in cells treated with F5/35‐ZD55‐IL‐24 (*P* < 0.05). F5/35‐ZD55‐IL‐24 + TMZ significantly increased Bax expression (*P* < 0.05) (Figure 6A). Our findings showed that a combination of F5/35‐ZD55‐IL‐24 and TMZ resulted in inhibition of Bcl‐2 and Pro‐caspase‐3, but increased expression of Bax (Figure 6A).

### 3.7 F5/35‐ZD55‐IL‐24 in conjunction with TMZ increased p53 but decreased MGMT expression

Previous research found resistance to alkylating agents such as TMZ to be associated with increased expression of the DNA repair protein, MGMT.^26^ In addition, p53 might be involved in triggering MGMT expression, because both p53 and MGMT are activated by DNA breaks. Therefore, we examined whether F5/35‐ZD55‐IL‐24 could regulate p53 and MGMT. F5/35‐ZD55‐IL‐24 induced greater upregulation of p53 expression and downregulation of MGMT levels than did ZD55‐IL‐24 (*P* < 0.05) (Figure 6B). The level of MGMT showed no significant difference between cells treated with F5/35‐ZD55‐IL‐24 alone and F5/35‐ZD55‐IL‐24 + TMZ (*P* > 0.05). The levels of p53 were higher in cells treated with F5/35‐ZD55‐IL‐24 + TMZ than in cells treated with F5/35‐ZD55‐IL‐24 alone (*P* < 0.05); however, there was no difference between cells treated with TMZ alone and PBS. These results indicated that TMZ did not upregulate p53 expression or downregulate MGMT expression. The combination of F5/35‐ZD55‐IL‐24 + TMZ led to downregulation of MGMT and upregulation of p53.

### 3.8 In vivo antitumor effect

**Figure 7:**
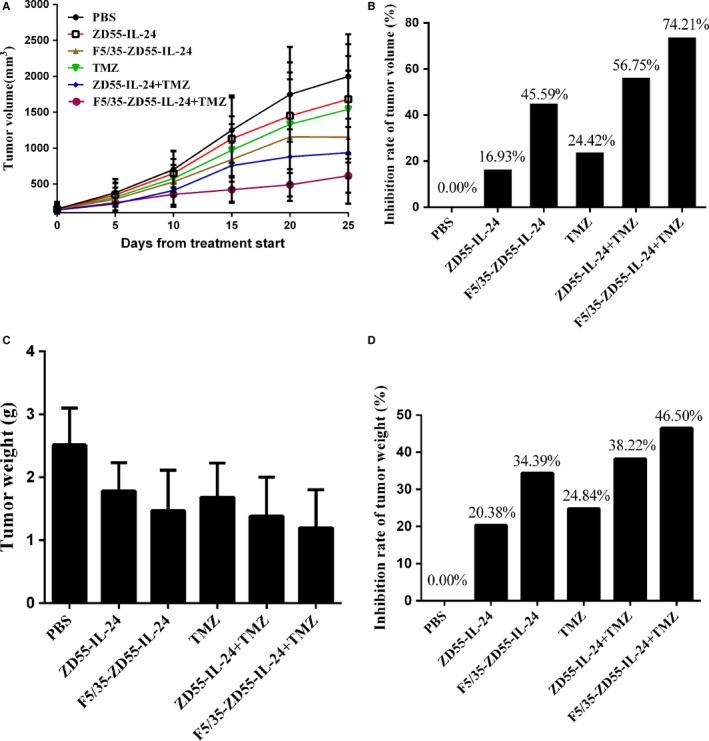
In vivo antitumor effect. A, Tumor volume changes induced by different treatments. B, Inhibition of the increase in tumor volume. C, Tumor weight changes induced by different treatments. D, Inhibition of the increase in tumor weight

The antitumor effect of F5/35‐ZD55‐IL‐24 in conjunction with TMZ was assessed in vivo. We found F5/35‐ZD55‐IL‐24 in conjunction with TMZ significantly inhibited tumor growth (Figure 7A). In addition, the reduction in the tumor volume was largest in the F5/35‐ZD55‐IL‐24 + TMZ group (Figure 7B). Similarly, after all treatments, the tumor weight was obviously reduced by F5/35‐ZD55‐IL‐24 in combination with TMZ (Figure 7C). Tumor weight was reduced by 46.50% in the F5/35‐ZD55‐IL‐24 + TMZ group, which was the largest reduction achieved among all treatment groups (Figure 7D).

### 3.9 Immunohistochemistry for IL‐24, MGMT, Bcl‐2, and Bax protein levels in vivo

**Figure 8:**
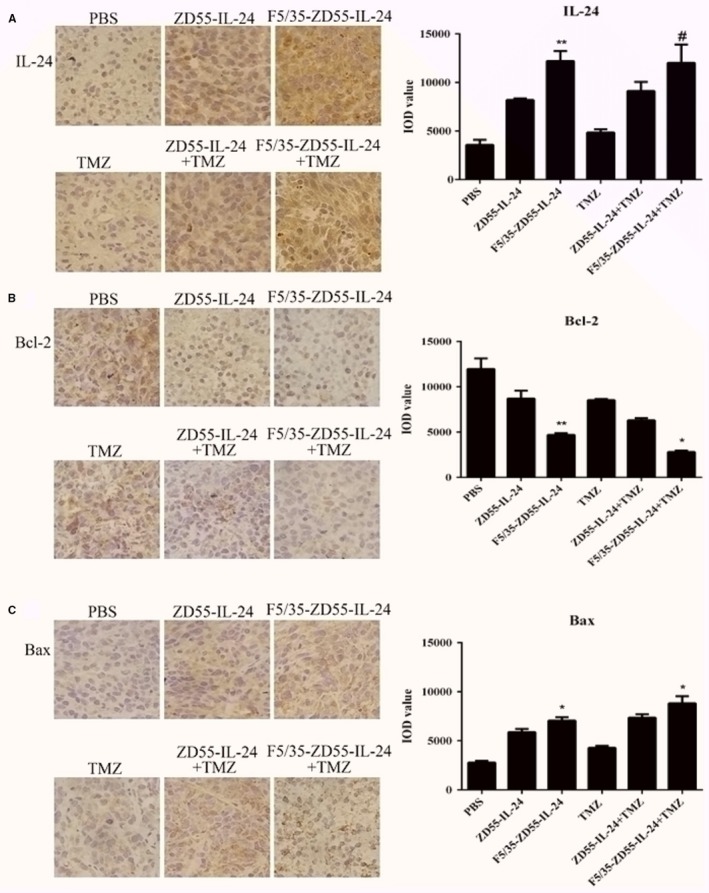
Immunohistochemistry for IL‐24, Bcl‐2, and Bax protein levels in vivo. A, Immunohistochemistry and quantification for IL‐24. B, Immunohistochemistry and quantification for Bcl‐2. C, Immunohistochemistry and quantification for Bax. Original magnification, 400× (scale bar, 25 μm). IOD: integral optical density. *P < 0.05 and **P < 0.01, F5/35‐ZD55‐IL‐24 vs ZD55‐IL‐24, F5/35‐ZD55‐IL‐24 + TMZ vs F5/35‐ZD55‐IL‐24; # *P* > 0.05 vs F5/35‐ZD55‐IL‐24

We used immunohistochemistry to detect IL‐24 protein expression in vivo. The results showed that F5/35‐ZD55‐IL‐24 and ZD55‐IL‐24 could stably mediate IL‐24 expression in vivo, and the level of the IL‐24 protein in the F5/35‐ZD55‐IL‐24 group was higher than that in the ZD55‐IL‐24 group (*P* > 0.05). However, there was no significant difference between the F5/35‐ZD55‐IL‐24 group and F5/35‐ZD55‐IL‐24 plus TMZ group (*P* > 0.05) (Figure 8A).

The anti‐apoptotic protein Bcl‐2 and pro‐apoptotic protein Bax were also detected using immunohistochemistry (Figure 8B,C). We found that F5/35‐ZD55‐IL‐24 and ZD55‐IL‐24 could significantly upregulate the level of the Bax protein and downregulate that of the Bcl‐2 protein compared with those in the PBS group, and the effect in the F5/35‐ZD55‐IL‐24 group was more obvious than that in the ZD55‐IL‐24 group (*P* < 0.05). Moreover, the F5/35‐ZD55‐IL‐24 + TMZ group and ZD55‐IL‐24 + TMZ group upregulated Bax protein and downregulated Bcl‐2 protein more significantly than did the TMZ alone group. However, the effect was the most significant in the F5/35‐ZD55‐IL‐24 + TMZ group.

## 4 DISCUSSION

Temozolomide has a fairly low response rate in melanoma, <20% of patients receive a very limited survival benefit from it, because of chemoresistance.^27^ Mechanisms of this chemoresistance have been described, including impaired drug transport, multidrug resistance‐associated protein, detoxification, or enhanced DNA repair. Panagiotis's team showed that combination therapy with a proteasome inhibitor (bortezomib) and TMZ could increase the death rate of U87 glioma cells compared with that induced by monotherapy.^28^ Egger et al^29^ reported a combination therapy of adenovirus (Ad‐FKHRL1/TM) and TMZ, which resulted in a greater tumor‐killing effect in vitro and in vivo than for either therapy alone. Thus, combining different types of antitumor therapy with TMZ might be a new approach to improve the response rate of melanoma cells to TMZ.

As novel antitumor therapeutic agents, CRAds not only selectively replicate in and lyse tumor cells, but can also amplify the expression and efficacy of therapeutic genes.^14^, ^15^ IL‐24 can suppress cancer cell growth and induce apoptosis and is therefore commonly used in tumor gene therapy.^30^

Our group previously found that delivery of IL‐24 via a CRAd (ZD55‐IL‐24), in combination withdacarbazine, has a greater antitumor effect in melanoma cells than either of these agents when used alone.^30^ ZD55‐IL‐24 attaches to tumor cells using CAR; however, the expression of CAR in most types of cancer cells is low or absent.^20^ Therefore, IL‐24 expression levels following ZD55‐IL‐24 infection of cancer tumor cells are still insufficient.

CD46 has been identified as a cellular receptor for the majority of species B adenoviruses including Ad11, 16, 35, and 50.^31^ Hoffmann et al analyzed several primary melanoma lesions, and in contrast with CAR, CD46 was highly expressed in all tissue samples. In addition, they evaluated 22 different adenovirus types, among which Ad35 had the highest internalization efficiency in short‐term cultures of melanoma cells.^32^ Takagi et al^21^ showed that CD46‐overexpressing cells had a significantly higher response to Ad35 fiber modification of oncolytic adenovirus and could improve viral infectivity and enhance antitumor efficacy in malignant mesothelioma cells, especially in those with low CAR expression.

In the present study, we used the melanoma cell lines A375 and MV3, both of which have low CAR expression, but high CD46 expression. We then synthesized a fiber chimeric adenovirus vector, F5/35‐ZD55‐IL‐24, in which the fiber protein of serotype 5 adenovirus (Ad5) was substituted by that of serotype 35 adenovirus (Ad35). We demonstrated that F5/35‐ZD55 could mediate higher levels of transfection than Ad5 virus in human melanoma cell lines, as demonstrated by the higher intensity of F5/35‐ZD55‐EGFP fluorescence.

In the present study, we showed that the combination of TMZ with F5/35‐ZD55‐IL‐24 apparently inhibited the growth of melanoma cells compared with either treatment alone or ZD55‐IL‐24 in conjunction with TMZ. In A375 tumor‐bearing mice, we also found that the combination of TMZ with F5/35‐ZD55‐IL‐24 had a significant inhibitory effect on tumor growth. Interestingly, we found TMZ did not affect the expression of IL‐24. Using immunohistochemistry, we found that F5/35‐ZD55‐IL‐24 in combination with TMZ inhibited the expression of Bcl‐2, increased Bax, and upregulated pro‐caspase‐3, which suggested that F5/35‐ZD55‐IL‐24 in conjunction with TMZ could increase the levels of pro‐apoptotic proteins and decrease the level of anti‐apoptotic proteins in melanoma cells.

## 5 CONCLUSION AND PERSPECTIVES

F5/35‐ZD55‐IL‐24 could not only express IL‐24, but also showed more efficient transduction of cell lines with low or absent expression of CAR compared with that of the Ad5 vector. Additionally, this combination with chemotherapy could be further enhanced by the use of CRAd‐gene therapy. This allows for a high concentration of locally activated drugs and thus minimizes systemic toxicity. However, the cytotoxicity of CRAds and their pharmacokinetic characteristics make the relationship between CRAd‐gene therapy and chemotherapy more complex. Further research and continuing development of oncolytic virus therapy and molecular oncology will enable researchers to develop a new generation of safer and more effective strategies for CRAd‐gene therapy in combination with chemotherapy.

In summary, F5/35‐ZD55‐IL‐24 in combination with TMZ produced an additive or synergistic antitumor effect in vitro and in vivo. Importantly, replication of the virus in melanoma cells was not affected by TMZ. Thus, CRAds mediated gene therapy in combination with TMZ may have potential as an effective treatment for malignant melanoma.

## CONFLICT OF INTEREST

The authors declare that there are no conflict of interests.

## COMPLIANCE WITH ETHICAL STANDARDS

Animal welfare and the experimental procedures were carried out strictly in accordance with the ‘‘Guide for the Care and Use of Laboratory Animals.”^25^
